# Advances in automated real‐time flow cytometry for monitoring of bioreactor processes

**DOI:** 10.1002/elsc.202100082

**Published:** 2021-11-12

**Authors:** Anna‐Lena Heins, Manh Dat Hoang, Dirk Weuster‐Botz

**Affiliations:** ^1^ Institute of Biochemical Engineering Technical University of Munich Garching Germany

**Keywords:** automated flow cytometry, bioprocess monitoring, online flow cytometry, population heterogeneity, process control

## Abstract

Flow cytometry and its technological possibilities have greatly advanced in the past decade as analysis tool for single cell properties and population distributions of different cell types in bioreactors. Along the way, some solutions for automated real‐time flow cytometry (ART‐FCM) were developed for monitoring of bioreactor processes without operator interference over extended periods with variable sampling frequency. However, there is still great potential for ART‐FCM to evolve and possibly become a standard application in bioprocess monitoring and process control. This review first addresses different components of an ART‐FCM, including the sampling device, the sample‐processing unit, the unit for sample delivery to the flow cytometer and the settings for measurement of pre‐processed samples. Also, available algorithms are presented for automated data analysis of multi‐parameter fluorescence datasets derived from ART‐FCM experiments. Furthermore, challenges are discussed for integration of fluorescence‐activated cell sorting into an ART‐FCM setup for isolation and separation of interesting subpopulations that can be further characterized by for instance omics‐methods. As the application of ART‐FCM is especially of interest for bioreactor process monitoring, including investigation of population heterogeneity and automated process control, a summary of already existing setups for these purposes is given. Additionally, the general future potential of ART‐FCM is addressed.

**Abbreviations**
ART‐FCM: automated real‐time flow cytometry
FACS: fluorescence activated cell sorting
FC: flow cytometer

## 1 INTRODUCTION

In the past decade, flow cytometry (FCM) has proven to be an invaluable tool in clinical diagnostic as well as research [1]. It is a powerful high‐throughput method for rapid measurement of fluorescence characteristics of cells with single‐cell resolution while at the same time collecting information about the parent population the cells are randomly sampled from. This feature makes FCM the workhorse of single‐cell analysis [2, 3]. Especially, FCM is advantageous, when the characteristics of a significant amount of single cells are evaluated in several consecutive samples following a bioprocess. Furthermore, this method is generally faster than omics‐methods, particularly when temporal variations are of interest [4]. For detailed information about the function of FCM, the reader is referred to existing literature (for instance [3, 5–7]).

Nowadays advanced flow cytometers (FCs) allow to quantify up to 50 parameters for millions of cells at a speed of around 1000 events per second [8]. Thus, single cells in a bioprocess expressing fluorescent proteins as reporters for different cellular characteristics or alternatively single cells stained with one or several fluorescent dyes can be monitored simultaneously [8, 9, 10, 11, 12, 13, 14, 15, 16, 17, 18]. Also, the combination of reporter strain and dyes or cell size characteristics is applied [18, 19]. Its ability to measure single cell characteristics makes FCM also a suitable tool to monitor population heterogeneity in bioreactors as well as to investigate the cells’ physiological state under process‐related conditions [2, 7]. Additionally, FCM has become an essential tool for investigating population dynamics in mixed cultures and quantitative studies of microbial communities differentiating different cell types based on cell size, morphology or fluorescence properties [20]. As a consequence, multi‐dimensional datasets are recorded. These can be analyzed applying advanced algorithms adapted from research areas where FCM is already a standard analytical tool [8, 21–25]. Otherwise simple statistical tools can be applied to exploit the underlying cellular expression pattern in consecutive bioprocess samples [26].

Even though the data analysis methods for FCM samples taken from bioreactors are far from being on the level of instrument advances, automated procedures for sampling from the bioreactor and sample preparation which would supplement this versatile and fast method, are surprisingly largely unexploited [27, 28]. Especially, when considering the application of FCM for automated process control, bioprocess monitoring and optimization, the analysis is apart from some examples [1, 28–32] mostly done off‐line or at‐line [33] to the bioprocess. The main reason was suspected to be that a complex interface between the bioreactor and the FC is needed [28]. In contrast, in fields, like water analytics, online process monitoring with FCM is routinely applied [34, 35, 36].

Indeed, integration of online measurement would, apart from following the trend of digitalization, have several advantages [37]. A process could be “continuously” monitored without intense cost in labor and time, filling gaps between manual sampling intervals and generating detailed pictures of changes in cell population distributions with temporal resolution [27, 38]. Furthermore, an automated procedure would be more precise than manual sample handling. Additionally, time can be saved as traditionally several consecutive steps in manual sample preparation have to be performed prior to FCM measurement [38, 39]. Also, depending on the measurement capacity, process monitoring can be performed on two levels, revealing rapid changes of single cells, which can be challenging to follow manually and changes on bigger time scale with regular sampling over several days which makes FCM a flexible tool for bioprocesses with different organisms or process goals. Then, this method can also be applied for process control [2, 28] and optimization as the metabolic state and growth of the cells in the bioreactor can be rapidly assessed [40]. Afterwards, process conditions can be feedback regulated to be favorable for the majority of cells or to enrich cells with advantageous characteristics for the targeted process goal [41, 42, 43].

Before reviewing the parts, state‐of‐art, advances and challenges in introducing an automated FCM method into a bioprocess, the name of this method should be defined, as wording is used inconsistently. In literature terms like “online,” “automated” or “real‐time” FCM are found that do not always caption the automated method described above. Instead, sometimes the at‐line or off‐line measurement of consecutive samples is meant, during which the FC is not directly coupled to the bioreactor. “Online” or “continuous” FCM might not be accurate because samples are taken in a range of several minutes. Consequently, the picture is slightly shifted compared to what happens in the bioprocess at the moment of data visualization. For this reason, in the following the term automated real‐time flow cytometry (ART‐FCM) will be used.

## 2 COMPONENTS OF AN ART‐FCM

**FIGURE 1:**
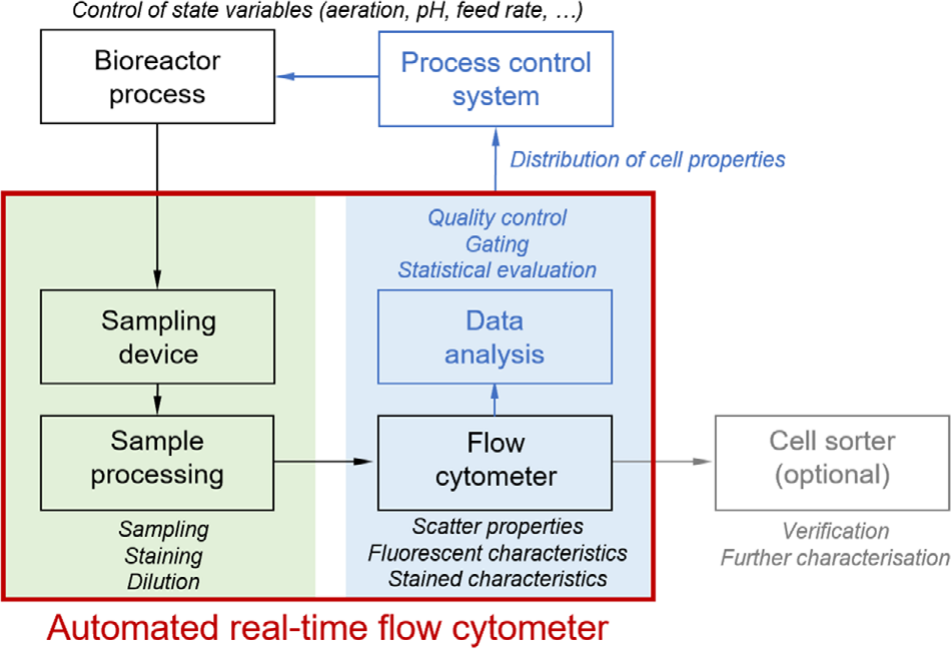
Overview of components of an automated real‐time flow cytometer and its application possibilities in bioreactor processes. The system can be divided into two general units: the sample preparation comprising the sampling device and the sample processing step(s) (green background) and the sample analysis that includes the flow cytometer itself including sample delivery and the (automated) data analysis (blue background). Both units are interconnected as they represent consecutive steps

ART‐FCM for automated monitoring requires additional parts compared to conventional at‐line or off‐line FCM. The first automated systems for real‐time assessment of the dynamics in the physiological state of cells, applied flow injection analysis coupled to FCM [35, 44, 45] and were often simple compared to modern systems (summarized in Table 1). However, the general setup still compromises the same basic units, sample preparation (sampling device and sample processing) and analysis (sample delivery to the FC, the FC and the (automated) data analysis) (Figure 1). Sometimes also specialized algorithms are applied that store samples in a specific format or do the pre‐treatment for subsequent automated data analysis and potentially feedback control of the experimental device. However, this part is so far rarely established [28, 46].

**TABLE 1:** Overview of automated real‐time flow cytometers and their components that were built and employed in published studies sorted by research area where the respective system was first deployed

References	Flow cytometer/FACS	Components				
		Sampling from external device	Staining/dilution	Temperature controlled	Automated data analysis	Sampling frequency
Systems developed for water analytics						
[34], [36], [49], [72]	BD Accuri C6	X	X	X	(X)	1‐15 min
[50], [57]	CytoBuoy	X	–	–	X	5 min
Systems developed for bioprocess monitoring						
[38], [39]	Beckman Coulter Cell Lab Quanta SC	X	X	X	–	24 h
[28], [29], [46]	BD Accuri C6	X	X	X	–	15‐60 min
[54]	Partec CyFlow Space	X	X	X	–	3‐4 min
[1], [53], [55], [56]	BD FACSCalibur, Guava easyCyte	X	X	X	(X)	15 min
[31]	Ortho Cytofluorograf IIs	X	X	X	(X)	3‐4 min
[27]	BD Accuri C6	X	X	X	–	<1 min
Systems with autosampler/pipetting roboter						
[66]	BD FACScan	X	X	(X)	X	20 min
Other systems						
[62]	Coulter Elite	X	X	–	–	<1 min
[61]	BD FACS Analyzer	X	X	X	–	<1 min
[51]	BD FACS Analyzer	X	X	–	–	2‐5 min

References are listed in alphabetical order.

### 2.1 Sampling device

In ART‐FCMs, the sample is mostly withdrawn from a bioreactor by means of an automatically controlled peristaltic pump, optionally filtered and transferred into sample processing units of different kind [27, 28, 34–36, 47–49, 50]. In that way, automated sampling can be performed every 3–15 min including 2 min measuring time followed by 1 min rinsing and preparation for the next sample [48, 51]. If the sample is stained and subsequently incubated, sampling with ultra‐high (1 min) and high (15‐20 min) temporal resolution to investigate short‐term microbial dynamics from up to three bioreactors for periods over 24 h, respectively, up to 2 weeks is possible [28, 34, 36, 48, 52]. Different systems are used for high and ultra‐high temporal resolution of sampling, simplifying the steps for sample processing when following short‐term microbial dynamics [27, 34, 35, 48]. Advantages of this system are that it can be run dis‐ or continuously with user defined frequency, is technologically simple and can flexibly be connected to various sample processing units. Disadvantageous is, however, that the system is prone to clogging because no cleaning procedure is implemented. This might also lead to carryover between subsequent samples.

Another system for automated sampling from bioreactors is a sample loop that is reconnected to the bioreactor via a peristaltic pump [1, 30, 31, 53], which continuously withdraws sample. In the loop, the sample passes through an optional degassing or de‐foaming unit to prevent disruption of the operation of the sampling device by trapped air bubbles [54]. Depending on if it is time to sample, the sample is either rapidly re‐circulated to the bioreactor, minimizing; however, not avoiding, possible influences on cell physiology due to unequal conditions in the sampling loop and the bioreactor, or fed into a measurement line or a micro‐chamber for further processing [53, 54, 55, 56]. With a loop with degassing unit, that is additionally flushed after each sample, the minimum sampling interval was determined to be 5 min, which is long compared to other systems [57]. As the above described system, this system can flexibly be combined with sample processing units, while allowing continuous sampling with user defined frequency.

Another sampling device, originally developed for rapid sampling in large numbers and cell inactivation for analysis of intracellular components in continuous or fed‐batch processes with *Escherichia coli* and *Saccharomyces cerevisiae* [39, 58–61], was also adapted for ART‐FCM of Chinese hamster ovary (CHO) cell cultures [38, 39]. Employing the device, samples were taken in less than 0.2 s during up to 2.5 days or every 30 s to 5 h for experiments up to 1 week, opening up broad application possibilities. Additionally, the device is fitted to standard port dimensions of laboratory scale bioreactors and can therefore be flexibly connected. For sampling, a programmable valve automatically opens after fixed time intervals [38, 39, 62, 63]. Then, the sample is either directly withdrawn [62] or the sampling pipe firstly flushed, which avoids, together with the automatic cleaning between subsequent samples, carryover between samples and lowers the contamination risk [38, 39, 60, 63]. However, additional flushing and rapid sampling can lead to volume variations in the bioreactor, which in the end might have an influence on process physiology.

In another setup, samples for ART‐FCM are taken from cultures grown in micro titer plates of 400–600 μL working volume inside a robotic platform using an automated pipetting device [64, 65, 66]. Samples are transferred to new plates for optional dilution with buffer before the whole plate is moved into the FC. In this way, 10–100 μL sample of up to 96 cultures can be withdrawn every 10–20 min for around 15 h. Consequently, this setup does not only allow high‐throughput, but also a high degree of parallelization. To account for unavoidable volume loss due to sampling, each vial has to be regularly refilled with fresh medium [64]. Nevertheless, this system is only suitable for short term experiments and highly technologically demanding compared to other sampling devices.

Therefore in conclusion, the three presented simple systems seem better suited for bioprocess monitoring, also considering the investment costs. Comparing them, possible influences on cell physiology found when applying the second system should be avoided. Therefore a system appears most reasonable that combines the standard bioreactor port compatibility and possibility to clean of the third system with the flexibility of the first system.

### 2.2 Sampling processing

After sampling, the sample is mostly not directly transferred to the FC but further processed, for instance diluted with sheath fluid or buffer, stained or mixed with a reacting agent. For this purpose, specific interfaces have been developed and partly commercialized [2, 34].

In simple systems, working by the in‐line mixing principle, fluids for dilution or reacting agents of different kind are introduced through a controllable multi‐way valve into the sample line [10, 28, 36, 38–39, 40, 44, 51, 63, 66]. Instantaneous inline mixing of sample and reagent is enabled by equipping the reaction line with mixing or vortexing devices or by injection of sterile air [35, 61, 67]. Afterwards the sample is incubated by continuously flowing through an often temperature controlled line of defined length and volume, which allows to precisely define the contact time [34, 35, 48, 68]. Considering incubation for the dye to react, a continuously withdrawn sample could be stained every 5–15 min with SYBR Green for evaluation of DNA/RNA content [34, 36, 38, 39, 48]. Dilution can also be performed in these systems [10, 28] adjusting the flow rates of the sampling pump and a pump for addition of sheath fluid to yield user‐defined dilution factors. A disadvantage of this system is, that it is impossible to perform sample processing tasks in parallel. In addition, depending on the tasks, the long reaction line prolongs the processing time, even if all parts are placed in close proximity. Moreover, this system needs precise characterization, calibration of the dilution system and strict harmonization of the action of all parts to ensure a robust staining and measurement procedure. Due to its complexity, the tube system should be checked for possibilities of clogging, fouling or sedimentation of cells.

Other systems include an air‐bubble free, stirred micro‐chamber that can be flexibly applied for performance of diverse tasks such as dilution, fixation, staining, mixing and washing [1, 31, 51, 54, 68]. A chamber facilitates mixing in down to 1–2 s in a predictable manner [31]. Moreover, addition of liquid of different kind is controlled by connecting the micro‐chamber to a multi‐way valve [31, 51, 62] delivering distinct liquid volumes with reproducible timing [51, 68]. In this way, unwanted side‐reactions are avoided as separate flow lines for sample, reagents and carrier solution can be implemented increasing the accuracy of the performed reactions [31, 68].

A special feature of these systems is, that it is possible to easily determine cell concentrations in the incubation line [1]. However, for concentrated cell samples above 2.0 × 10^6 ^cells mL^−1^, accuracy decreases as it is impossible to distinguish every single cell.

Micro‐chambers can also be employed for dilution of samples by addition of buffer or sheath fluid with defined flow rates until the cell concentration drops to a desired value that is approximated by the previous sample or specific number of events in the FC [1, 30, 54]. There is a risk of excessive increase in dilution factor by outlier samples, which were shown to have a detrimental effect on the counts in the FC throughout the remainder of an experiment. Therefore, a limit for dilution factor increase between subsequent samples should be defined [1].

Another simple, inexpensive mixing chamber was built of a disposable plastic cuvette directly placed at the inlet of the FC [69]. The chamber is pre‐pressurized, temperature controlled and magnetically stirred. Up to five tubes for liquid addition from different reservoirs can be connected to the port head. Due to short distances, the time delay between sample preparation and measurement is reduced to a few seconds enabling measurement with high temporal resolution. Even though this system covers the same features as more robust micro‐chambers, it bears a risk of fatigue of material, so that it is reasonably applied only in short term experiments.

Another simple system employed a home‐built temperature controlled coaxial jet flow mixing device consisting of two capillaries. The sample is introduced through the inner locatable capillary, while the outer capillary is fixed and contains reagent. When sample and reagent get in contact they mix instantaneously due to different shear stress profiles inside the capillaries. Displacement of the inner capillary allows for variable contact times between sample and reagent that both flow with constant motion [61]. This system is limited to performance of a single task: mixing. Moreover, for accurate results, intensive characterization and calibration is needed, making it unfavorable compared to other devices presented here.

Comparing the described devices, a setup with non‐disposable micro‐chamber seems most flexible to perform diverse tasks and therefore best suited when building an ART‐FCM.

### 2.3 Delivery of the sample

After processing, the sample has to be delivered and loaded into the FC. Thereby the first challenge is to establish a connection between FC and sample processing device, as they have potentially quite different requirements concerning pressure, material and stability [51, 68].

In simple cases, the sample uptake nozzle head of the FC is attached to a tube with a stem at one side to which the tubing of a sample line is directly attached [1, 38, 39, 62, 65]. Samples are then pumped to the FC, limiting carryover between samples as liquid can only move in one direction. Often, the passage is further restricted by the action of a remote controlled valve [1, 28–30, 54, 55, 70, 71]. The sheath fluid is driven by the pressure regulation system of the FC. For this purpose, the line that normally pressurizes the injection tube of the FC is connected to the sheath fluid reservoir. With this simple setup the normal action of the FC is not disrupted, however there is a high risk of technical failure, especially considering leakage.

As every FC is equipped with a flow cell for loading samples into the instrument, tubing can also directly be attached to the flow cell [31, 34, 35, 48, 50, 51, 57, 68, 72]. A valve, which is only opened when a measurement is about to take place, then separates the flow cell from the sample line. After measurement, the sample is removed to a waste container, allowing inline cleaning prior to measurement of the next sample. At the same time, sheath fluid is automatically refilled so that the pressurized system of the FC is not influenced [1, 30, 36, 73]. As above, there is a risk of technical failure and also clogging of tubes might be a problem.

In a special case, the flow cell of the FC was exchanged by a coaxial flow mixer which was connected to the FC via a modified sample nozzle head as new sample introduction nozzle [61]. A similar system, using a plastic flow cell directly placed at the injection port, is pre‐pressurized by the pressure line of the FC to ensure compatibility with the flow requirements inside the FC [69]. These systems allow measurement with high temporal resolution, but they are less flexible to be connected to different FCs and the plastic cuvette seems to be less durable.

Some systems, in which samples are transferred from for instance micro titer plates [66], use an auto‐sampler with a robotic arm as interface to the FC. As mentioned earlier, this system is only suitable for short term experiments, technologically demanding and involves high costs. Consequently, a setup where the tubing is directly attached to the flow cell of the FC and that includes cleaning after each measurement seems best suited.

### 2.4 Measurement of sample/sampling process control

Normally, systems are fully automated so that the action of injectors, valves, pumps and the stirrer of the mixing chamber are controlled by custom‐made LabView, C++ or Matlab routines. These often run on a personal computer equipped with a data acquisition card on which also the data acquired by the FC internal software are processed [1, 31, 34–36, 46, 48, 51, 54, 60, 63, 64, 73]. The computer that coordinates the complete systems operation can be operated via remote access allowing unlimited data access and transmission rates with high location flexibility [1, 50].

Mostly, automated measurement is triggered by loading the sample into the FC [34–36, 48, 73] while at the same time sensors embedded in each piece of the system report their state to coordinate the measurement procedure [64]. The initiation of the procedure can be coupled to constraints for instance, that measurement can first happen after full cleaning of all lines to avoid cross‐contamination between successive samples or appropriate dilution of the sample [10, 28].

After measurement, feedback on cell numbers can be given to the respective parts of the ART‐FCM for automated calculation of an appropriate dilution factor for the subsequent sample [1, 54, 56]. Additionally, for instance the growth rate of the culture as well as other parameters can be determined and visualized automatically as the experiment progresses [54].

Also sheath fluid is automatically, continuously replenished ensuring that the system can be operated self‐sustained for several days without interruption or maintenance [1, 34, 35, 48]. Consequently, bioprocesses can be monitored without supervision for up to 14 days, sampling every 1‐60 min depending on the organism, the mode of operation and the system configuration, revealing FCM data with high temporal resolution [1, 10, 28, 29, 31, 40, 46, 52, 61, 70, 72]. The gap between subsequent samples is dependent on which tasks have to be performed next to the actual measurement of the sample including flushing, staining or dilution, sample transfer as well as data transfer and analysis [46, 50, 57]. To avoid contaminations, ART‐FCM systems are at least daily rinsed with detergent containing hypochlorite [34, 35, 38, 48–50] and all connecting parts are cleaned in between different experiments [46].

The FCM measurement mostly uses a blue 488 nm laser with a voltage of 10–100 mW and data collection with one or more band respectively long pass filters of different wavelengths (typical filters: green: 520–533 ± 20–30 nm, orange: 585 ± 40 nm, red: 610 ± 30 nm and deep red: <670 nm) depending on how many fluorescence properties are accessed in parallel [27, 31, 35, 36, 38, 39, 54, 55, 66, 72, 73]. Also collection of side scatter (SSC) and forward scatter (FSC) revealing information on cell morphology and size is common [29, 36, 50, 54, 56, 57, 73]. In advanced experiments, further lasers are applied [27].

Measurement is normally done for 30–90 s at a fixed flow rate of 16–66 μL min^−1^ for samples from bioreactors using either the unlimited run function of the FC or automated activation for each sampling. The threshold value for sample recognition and discrimination from background noise of the instrument or the medium is mostly set in one fluorescence or scattering channel [10, 27, 28, 34, 35, 46, 48, 52, 64, 72]. Depending on the organism to be measured, predefined linear respectively logarithmic amplification is used [27, 31, 36, 38, 39, 50, 54, 57, 71, 74]. Electronic gates are used to discriminate specific subpopulations in all collecting channels, for instance cells with positive and negative fluorescence level [27, 36, 38, 39, 48, 72, 73]. Depending on the measurement accuracy targeted, 20,000‐300,000 events for bacteria and yeasts, and 5.000‐30.000 events for mammalian cells, respectively, [38, 39, 70] of one sample replicate are collected at rates of 50–1,000 events per second [10, 27, 46, 52, 54, 74]. For this purpose, cells are diluted accordingly, to avoid signal distortion by high sample concentrations [29].

When spectral overlap of fluorescence properties occurs in multi‐color experiments, automatic compensation, offered by most FCM software tools, can be applied using the first samples of the culture before the experiment is initiated [30, 38, 39, 75–77]. Afterwards, all instrument settings are kept unchanged to achieve comparable data [34, 35, 48, 72].

After measurement, the recorded data are collected by proprietary software of the FC or custom‐made routines and stored as list mode data files such as fcs or csv [1, 34–36, 46, 48, 73]. Specialized software or scripts are used for subsequent automated real‐time data transfer, storage and analysis [27, 36, 50, 57, 60, 66, 72, 73].

### 2.5 Automated data analysis

To exploit the full potential of ART‐FCM, the implementation of automated data treatment methods to display data in real‐time and visualize temporal shifts of specific parameters is wishful. Furthermore, automated data analysis can safe a tremendous amount of time as it is more efficient than manual, off‐line evaluation of samples [78]. Automated methods might be adapted from immunological studies because these experiments also generate large multi‐dimensional data sets with temporal resolution [22, 78, 79]. Furthermore, recently supervised and unsupervised algorithms for data visualization, quality control, automated gating as well as classification and identification of cellular populations have been developed (for a summary of R‐based algorithms and their function see Table 2). These were also topic of some review articles [8, 22, 23, 78, 80]. However, they have not yet made it into mainstream due to intrinsic complexity and lack of comprehensive and easy‐to use [78, 81].

**TABLE 2:** Overview of R‐based algorithms for automated data treatment that could be adapted for an automated real‐time flow cytometry setup

Method	Description	Reference
Data quality		
flowAI	Cleans flow cytometry files from anomalies during measurement procedure	[87]
Data visualization		
flowFit	quantitative analysis of cell proliferation in tracking dye‐based experiments after gating	[88]
flowViz	plots flow cytometry data in different layers avoiding information loss	[89]
ggCyto	Algorithms based transformation of data and axes and visualization according to specific structures	[86]
SCENERY	Web server featuring several standard and advanced cytometry data analysis methods	[81]
Automated gating		
Supervised		
flowPeaks	Gating of high‐dimensional data, identification of irregular shape clusters	[96]
flowDensity	Gating analogous to a manual gating strategy based on data density clouds	[79]
OpenCyto	Hierarchical automated gating	[91]
DeepCyTOF	Deep learning algorithm for automated gating	[92]
GateFinder	Gating by stepwise creating two‐dimensional convex gates of best fit	[93]
Semi‐Unsupervised		
flowLearn	Gating combining flowDensity with a deep learning algorithm	[94]
NetFCM	Gating combining clustering and principal component analysis	[95]
Unsupervised		
flowMeans	Gating based on K‐means	[98]
SPADE	Gating based on hierarchical clustering	[100]
Citrus	Gating based on hierarchical clustering	[101]
flowPeaks	Gating based on K‐means and finite mixture modeling	[96]
FLAME	Gating based on finite mixture modeling	[97]
Hypergate	Gating via a best fit hyperrectangle	[99]
Automated identification and classification		
CHIC	Grey scale images are automatically processed and batch‐wise compared	[108]
CyBar	Following manual gating, a mask compromising all gates of all samples is compared within a batch	[107]
FlowFP	Uses probability distributions functions to equal sized bins that are combined to a template	[104]
Dalmatian Plot	Black and white images of manually gated samples automatically processed via images analysis	[106]

Analysis of FCM data mainly takes place in R or MATLAB. Whereas R is more common and algorithms and plotting tools are more advanced (see Table 2), as it is historically used for analysis of immunological samples, statistical files are often generated in MATLAB [82, 83]. For both tools standardized functions to load FC data as FCS files exist. For MATLAB the fcs_read and fcs_readfcs algorithms are popular [19, 26, 52, 84, 85]. For R, the Bioconductor platform exists, that hosts the largest collection of open source FCM software covering data analysis and visualization of FCM data [86]. It also includes the flowCore package with functionality to import FCS‐files. Another R‐based webserver, Single CEll NEtwork Reconstruction sYstem (SCENERY), provides options for data (pre‐)processing, visualization, statistical analysis and modelling [81].

#### 2.5.1 Quality control and data visualization

Prior to detailed analysis, acquired data should be quality controlled for unwanted events to avoid interference and improve reliability of automated data analysis. For this purpose, automated algorithms exist, like flowAI [87], which can automatically detect and remove anomalies during the measurement procedure in the FC. This includes instability of signal acquisition as well as outliers and margin events at the limits of the dynamic range [87].

First step of an automated data analysis procedure could be generation of histogram or scatter plots of events in relation to a particular channel or multiple channels stacked offset with timely resolution and display them in real‐time [8, 33, 46, 54, 64]. In R, specialized packages for data visualization exist, for instance ggCyto [86], flowFit [88] and flowViz [8, 89] (see Table 2). However, taking full advantage of the underlying information in the data is often prevented by complexity of the analysis with the gating step as major bottleneck [8].

#### 2.5.2 Automated gating

Automated gating can objectively define subpopulations, account for population overlap due to measurement uncertainty and replaces subjective, time‐consuming and inaccurate manual gating [21–23, 78, 80, 90]. Available methods for automated gating were critically assessed and found to be sufficiently mature to be reliably applied [21]. Some methods even enabled to discover cellular populations that were unexpected or non‐evident to experimenters [80]. However, they are not yet well accepted and known [78].

Automated gating is performed based on supervised or unsupervised mathematical modelling of fluorescence intensity distributions of different cellular characteristics, so far exclusively applying R‐based algorithms (see Table 2). Supervised algorithms need a training data set defining classes respectively populations that each cell event belongs to [8]. The algorithm will learn this information during training stage and later apply it to assign unlabeled events to one pre‐defined class. Examples for these algorithms are flowDensity [79], OpenCyto [91], deepCyTOF [92] and GateFinder [93].

The semi‐supervised algorithm flowLearn [94] uses a few manually gated samples to fast and accurately predict gates on other related samples through density alignments. Another semi‐supervised approach, NetFCM, applies a combination of clustering and principal component analysis to mimic manual gating and quantify differences between samples [95].

Unsupervised algorithms function without user input. Mostly they define clusters based on similarity of events, meaning clusters contain events that are more similar than events of another cluster [8]. Clusters can be identified model‐based, applying for instance a Gaussian mixture model [24], like the flowPeaks algorithm [96] and the software tool FLAME [97], or non‐model‐based using flowMeans or Hypergate [98, 99] (see Table 2). Further examples are Spanning‐tree Progression Analysis of Density normalized Events (SPADE) [100] and Citrus [8, 101] that both identify cell populations based on hierarchical clustering. Other tools for automated gating apply probability state modelling (PSM), which bears great future potential as it works operator independent, includes quality control and exhibits a high level of objectivity, speed and precision [90, 102].

#### 2.5.3 Statistical assessment of (sub)‐populations

After gating, (sub‐)populations can be statistically assessed to objectively describe temporal changes in their shape and intensity. Common are determination of mean fluorescence intensity respectively normalized mean, mode, median and the coefficient of variance, which can be related to noise in gene expression for fluorescence expressed together with cellular markers [26, 27, 52, 82, 103]. Width at baseline level, skewness and the slope of the cumulative distribution function plot can further quantify the shape of fluorescence distributions [26]. Where significant subpopulations appear, its percentage can be computed [19]. Calculations can be performed by MATLAB and R‐routines and the results automatically displayed in real‐time [26, 46, 54, 64]. Also a summary of the statistical assessment can be generated [1, 10, 28, 64, 66].

#### 2.5.4 Identification of microbial diversity in cell communities

Multi‐parameter cytometric histograms combined in one data set represent individual, unique fingerprints of microbial communities at certain time points under defined environmental conditions [20, 25, 72]. Therefore, cytometric fingerprinting is emerging as powerful, high‐throughput tool to robustly analyze bacterial populations and monitor microbial diversity across phenotypical characteristics [25, 104, 105]. It reveals results faster than the corresponding molecular biological tools. Consequently, it bears great potential for real‐time in‐situ monitoring of microbial diversity with ART‐FCM [82, 83]. In short, the method compromises analysis of distributions of different physiological traits that explicitly characterize certain phenotypes [82].

Next to manual methods [72], several automated tools for cytometric fingerprinting have been developed, like the semi‐automated method Dalmatian Plot [106] and Cytometric Barcoding (CyBar) [107], and the automated methods Cytometric Histogram Image Comparison (CHIC) [108] and flowFP [25, 109] (see Table 2). These methods were found suitable to monitor structural changes in microbial communities comparing sensitivity, required experience of the operator, time demand and software requirements [25]. Manual gating steps could potentially be replaced by methods for automated gating introduced above. Another method [110] allows to estimate phenotype specific diversity metrics of the cytometric fingerprint and was applied to discriminate among 29 *Lactobacillus* strains and different growth phases of a microbial culture [49].

### 2.6 Fluorescence‐activated cell sorting

Fluorescence‐activated cell sorting (FACS) is a technique enabling sorting within the FC, thus offering possibilities for further analysis of (sub‐)populations of microbial cells with specific properties of interest [111]. In combination with high‐throughput screening, given by the FC, it can be utilized in versatile fields of research, however, traditionally, FACS is often used in diagnostic applications [112]. Due to its suitability for single‐cell sorting, it also allows isolation of rare cells such as stem cells, bacterial species or circulating tumor cells [113, 114, 115]. Moreover, FACS can be utilized for the screening and selection from large pools of variants as during directed evolution. Due to the ability of some systems for non‐destructive sorting, variants of interest can also be isolated. This provides intact cells for further rounds of screening, making FACS an attractive platform for iterative optimization of biomolecules [116, 117, 118, 119, 120].

Furthermore, in combination with single‐cell omics, currently being one hot research topic, however still very labor intensive, FACS could play a significant role in the future [121, 122]. Sorting of subpopulations prior to omics analysis enables ultrasensitive measurements. Thus, stochastic average masked signals of heterogeneous populations can be avoided. Consequently, the level of biological noises could be controlled and clear correlations could be established [123, 124, 125, 126, 127].

Another approach to monitor single‐cell physiology is the utilization of fluorescent reporter strains. When their fluorescence is analyzed by FCM, whole populations in bioprocesses can be screened regarding specific phenotypes [83]. ART‐FCM analysis could in the future be combined with FACS [128, 129] to an enhanced bioprocess monitoring system. It would not only enable the automated real‐time analysis of subpopulation formation throughout a process. Also isolation and subsequent enrichment of advantageous subpopulations for detailed characterization would be possible providing deeper insights into the sources of population heterogeneity.

#### 2.6.1 Implementation of FACS into ART‐FCM

Integration of FACS into ART‐FCM (ART‐FCM/FACS) is generally feasible because FACS systems and FCs share the same basic setup [130]. In comparison to FCs, however, the liquid jet, in which cells are singularized via hydrodynamic focusing, is in most FACS setups broken down to single cell encapsulating droplets. These droplets get electrically charged by voltage pulses and subsequently deflected by an electrical field underneath the flow cell. Yet so far, sorting in tubes, micro‐titer plates and even agar plates is possible [131, 132, 133].

In fact, the cell‐sorting procedure requires a nozzle, deflection plates, an output collector and cameras to ensure correct sorting. All these components are installed underneath the flow cell leading to no conflict with FCM measurement [134, 135]. Considering existing deflection techniques using electric or magnetic fields [136, 137], this results in a variety of systems for simultaneous single‐cell analysis and sorting. For insights into the FACS principle, the review of Cossarizza et al. is recommended [111].

ART‐FCM/FACS would allow simultaneous instrument preparations prior to measurements. However, additional quality checks are mandatory for stable sorting over extended time periods. Among them is the regular measurement of the drop delay. By feeding the system with commercialized beads, it is possible to adjust the nozzle amplitude automatically ensuring regular sized droplet formation. Furthermore, correct sorting into the output collector, which is visually checked doing test sorts into different positions, is important. To be capable of sorting rare subpopulations during a bioprocess, it is crucial to define precise gating thresholds. When subpopulations are easily distinguishable, manual gating can be straightforward. However, with subpopulations that only differ marginally, methods for automated gating mentioned above should be applied. Recently an approach [138] for automated gating by machine learning, entitled CellSort, which could possibly be adapted for ART‐FCM/FACS, was published. CellSort is based on a support vector machine (SVM). Vectors were created out of a historical FCM dataset and used to train the SVM generating 5000 randomized data points of positive and negative populations. Performance of the SVM was validated achieving a true positive rate of over 60% and a false positive rate below 5% [138].

Additionally, several aspects need to be considered for stable and robust sorting with ART‐FCM/FACS throughout a bioprocess. First, it is only possible without movements of physical objects. Even micro shifts of the flow cell, deflection plates or output collectors lead to errors in automatic handling, which could result in a premature shutdown of the device. Second, physical properties, like temperature and pressure, of the sheath fluid should be kept constant as it was previously shown that alteration during measurements lead to poorer yields and sort purities [139, 140]. Therefore, tempering of sheath fluid is necessary, by for example cooling with Peltier elements [140]. Additionally, controlling the interior instrument conditions, like temperature and humidity, would support sorting stability. Thirdly, an output collector should allow collection of multiple samples. For automated real‐time monitoring during bioprocesses for at least 24 h, micro titer plates seem suitable. A 96‐well plate for instance would allow sampling in 15 min intervals for 24 h. If the sorting procedure is precise enough, even 384 well plates could be applied, either allowing faster intervals or sampling over longer time periods [141]. Fourth, storage inside the output collector should be possible. This implies cooling of the sorted cells to preserve their current metabolic activity: the cooler, the slower the physiological changes inside the cell [142]. This can be realized by cooling of the collector implementing customized sample holders respectively adapt them from automated liquid handling platforms. This would even allow cooling of samples at sub‐zero temperatures, thus, increase long‐term durability of cells [143]. Dependent on the cell treatment afterwards, liquid solutions such as media, cryo‐protective agents or lysis buffer could be added prior to or after sorting by the action of a robotic liquid handling system. Like that, storage conditions of the cells are enhanced compared to storage in pure sheath fluid [144, 145, 146] and even sub‐cultivation is possible. This would necessitate sterile sorting which could be realized by providing an aseptic working area as in commercial systems like BD Influx [147, 148].

#### 2.6.2 Potential challenges for establishment of an ART‐FCM with FACS

Although the implementation of ART‐FCM/FACS seems straightforward, plenty of challenges remain. Among them is the temperature setting for the sorting procedure, which is advantageous both for keeping sheath fluid properties constant and providing ideal sample storage conditions. However, tempering the whole FC interior appears inappropriate and currently no device with cooling of the sorting route is commercially available, probably due to technological complexity and high investment costs. And last, it is unclear which temperature prevents cell damage prior to sorting. Considering sample storage, lower ones are favorable. However, there are potentially negative impacts on components of the ART‐FCM/FACS, for instance freezing of components. Consequently, partitioned cooling seems more practical. Similarly to sheath fluid tanks, output collectors could be cooled by Peltier elements [143].

Another major issue is sorting stability. Thereby, clogging of the nozzle, which normally needs to be cleaned prior to each sort, is a bottleneck. Therefore, strategies are necessary for specific cleaning in place intervals. Furthermore, filter units should be implemented fitted to the nozzle diameter in order to remove coarsed particles [147, 149].

Finally, FACS systems tend to be very bulky [112]. This hinders flexible movement and limits the applicability for bioreactor processes at different locations. Thus, miniaturization of FACS respectively ART‐FCM/FACS should be taken into account. One interesting approach could be the usage of so‐called μFACS which comprise sorting of events on a microchip [112, 150, 151].

## 3 AUTOMATED REAL‐TIME FLOW CYTOMETRY FOR BIOREACTOR PROCESS ANALYSIS

So far ART‐FCM has only rarely been applied for analysis of bioreactor processes. However, the future potential is immense as FCM is already the workhorse of microbial single cell analysis and its applicability in industrial bioprocesses has been demonstrated [2]. It would circumvent extensive amount of sample handling when seeking for detailed monitoring of cell physiological characteristics in a bioprocess with high temporal resolution [1, 30, 38–40]. Consequently, the logic technological progression is full automation of all analysis steps. Furthermore, dynamic evolution of microbial stress resistance and adaption is still poorly described, which however could be done in this setup [2]. Additionally, the data sets derived through ART‐FCM could establish baseline data for cultivation systems, as well as allow sensitive recognition of daily variations and specific events that would likely be missed or miss‐characterized by infrequent sampling [34, 35, 36]. Many observations are also not resolvable at population level [152]. Importantly, results are available in real‐time enabling informed decision taking during a running process [7].

### 3.1 Monitoring of physiological state of cells

As a prerequisite for robust process performance, it is essential to follow single cell physiology during a bioreactor process including the percentage of viable cells. Most commonly viability is assessed applying exclusion dyes, such as propidium idodide (PI) or fluorescein isothiocyanate (FITC), that stain nucleic acids in cells whose membrane is destructed [10, 28, 30, 34, 38–40, 48, 53, 55]. Using ART‐FCM, PI‐staining was applied in batch and continuous cultures of *S. cerevisiae* and *E. coli* to assess changes in viability distributions measuring every 15 min for over 40 h [1, 28, 30]. Thereby, the dye concentration and the optimal contact time between dye and cells were established to be critical parameters for reproducible staining results. PI staining was also utilized to investigate the effect of acetate in lignocellulosic hydrolysates, which are feedstocks for industrial biofuel production with *S. cerevisiae* [53]. ART‐FCM measurements revealed that elevated acetate concentrations led to decreased specific growth rate, accumulation of cells in G1 phase of the cell cycle and increased cell size.

Frequently, a dual viability assessment combining PI with the nucleic acid stain SYBR Green is applied in ART‐FCM setups [27, 34–36, 48]. This assay was successfully applied in short‐term and long‐term processes analyzing every 5 min during 60 h as well as every 15 min for up to 70 days, respectively [34, 35]. It allowed sensitive detection of bacteria over a broad concentration range tracking both gradual and dramatic changes in natural water samples, in samples from a drinking water pilot plant and from pure bacterial cultures [36, 48, 73]. Some studies solely applied SYBR Green to discriminate bacteria from background [46, 49] or dual staining with PI and FITC labeled Annexin‐V to investigate apoptosis and necrosis in CHO cell cultures [38]. With FITC alone cell size changes triggered by ethanol and temperature dependence of constitutively expressed GFP in yeast fed‐batch processes could be evaluated [55, 153].

Additionally, the lipid stain Nile red or BODIPY 493/503 for visualization of storage compounds like poly(R)‐3‐hydroxybutyric acid in *S. cerevisiae* and *Cupriavidus necator* or the DNA content specific stain mithramycin A for evaluation of cell cycle progression in *S. cerevisiae* have been employed in ART‐FCM setups [31, 71].

### 3.2 Monitoring of cell abundance

In some applications it is interesting to identify abundant cell types in a bioreactor process revealing the fingerprint of culture dynamics [82]. Examples are algae cultures that are run under non‐sterile conditions or mixed cultures, in which the ratio between different organisms is unknown. To identify microbial cells for instance SYBR Green is applied [36, 73]. The highly sensitive method originates from monitoring of microbial growth in water treatment plants, where changes in water quality are crucial [36, 48, 49, 73].

Also natural fluorescence emitted by photosynthetic pigments in algae cultures or auto‐fluorescence can be monitored in bioreactor processes [50, 57]. Especially, large cultures of microalgae would benefit from on‐line monitoring to achieve process control [32]. Analysis of FSC and SSC of *Synechococcus* phytoplankton cultures as well as pigment fluorescence in their natural environment applying ART‐FCM revealed a detailed picture of abundance variations of phytoplankton that could not be covered otherwise [57, 63].

### 3.3 Monitoring of process performance

For robust and high‐yielding bioreactor processes, it is essential, to monitor process performance concerning product formation, growth and robustness of production hosts. Some studies already applied ART‐FCM, more recently measuring fluorescence from genetically modified microorganisms. Mostly fluorescence of reporter proteins is recorded that are expressed together with cellular properties of interest for instance cell growth, stress response or product formation [10, 28, 54, 55]. However, if the product is fluorescent itself, the measurement is highly facilitated [63]. Considering monitoring of product formation dynamics with ART‐FCM, often GFP expression is used as a reference [31, 54]. The distribution of GFP formation was broad for *E. coli* cells including non‐producer and cells that produced significantly larger amounts than the average cells. Similar results were found in *S. cerevisiae* cultures [1, 31, 54] as well as that constitutive expression of GFP is highly temperature dependent leading to oscillations [153]. ART‐FCM was also a rapid method to test promoter strength, plasmid stability and culture variability [30] demonstrating that small genetic changes could result in large variations in product formation. Expression of GFP tagged human membrane protein monitored with ART‐FCM in cultivations with *Pichia pastoris* [54] could aid in selection of highly productive, stable strains. Similarly, mammalian cell lines were screened for high‐producing cells [41] as well as more robust and acetate tolerant strains for high‐yielding ethanol production by *S. cerevisiae* from lignocellulosic hydrolysates, which contain acetate in growth inhibiting concentrations, could be chosen [53, 56, 154].

ART‐FCM can also be employed to study temporal changes in fluorescence of cells following an event of interest, for instance induction of the SOS response or growth initiation of stationary phase in *E. coli* cultures [27]. In these experiments, a trigger, e.g. ciprofloxacin to induce the SOS response, is added and the cells response collected during a fixed time period. This demonstrates the broad applicability of ART‐FCM and its ability to collect detailed, time‐resolved information on complex processes.

Next to product formation, growth and thereby biomass generation is of interest for ensuring efficient bioreactor processes. The entire growth curve of *E. coli* could be followed with ART‐FCM visualizing that a state of balanced growth is never reached [31]. Without transforming production hosts with a specific plasmid, cellular growth rates can be measured via ART‐FCM combining bromodeoxyuridine and PI‐staining to determine the proportion of cells synthesizing DNA, and the total DNA content, respectively, [40].

## 4 AUTOMATED REAL‐TIME FLOW CYTOMETRY FOR INVESTIGATION OF POPULATION HETEROGENEITY

Population heterogeneity refers to the unequal behavior of cells originating from isogenic cultures due to cell cycle progression, environmental influences or genetic differences [2, 33]. It is known to be omnipresent and recognized as major source of issues during development and optimization of bioreactor processes [28, 33]. Apart from few examples, currently at‐line FCM is used for analysis of heterogeneities in bioprocesses, often afterwards scaling down single cell variability data to averaged values [33]. Since population heterogeneity is highly dynamic exhibiting strong temporal shifts, applying ART‐FCM, especially in combination with automated data treatment, would significantly improve resolution of the collected multi‐dimensional data [30, 33].

Many experiments investigating population heterogeneity apply reporter strains to follow population dynamics [33]. In these strains, fluorescent proteins are expressed together with a physiological characteristic of interest so that its evolution can be followed on single‐cell level. Applying *E. coli* and *S. cerevisiae* reporter strains constitutively expressing GFP together with PI staining and FSC measurement, dynamics in single cell growth, viability and cell size in batch cultures in a stirred‐tank bioreactor (STR) could be monitored by ART‐FCM [1, 30, 31]. Two subpopulations (active vs. less active cells) appeared, especially at the onset of stationary phase. The reason was probably decreased nutrient availability and a shift in metabolism which partially caused loss in cellular activity. Comparing the expression of different GFP variants in the same setup [30], three distinct subpopulations for GFP fluorescence, whose ratio changed according to growth phase, and significant population heterogeneity was found.

Other studies with ART‐FCM examined heterogeneity in GFP formation by an exponentially growing *E. coli* population, [31] respectively, production of GFP tagged human membrane protein from an alcohol oxidase promoter during *Pichia pastoris* cultivations in a loop bioreactor [54]. Initially, the populations were, apart from a minor fraction of “leaky” un‐induced cells, non‐fluorescent. After induction with IPTG respectively methanol, fluorescence increased, apart from a subpopulation that stayed non‐fluorescent. In addition, a subpopulation of high producing cells that could express more protein of interest within shorter time, probably due to a higher copy number, was found. Towards the end of the process, the majority of cells were unable to further increase expression levels, because they were fully loaded with peroxisomes. These findings might help in the selection of high producing, stable strains, especially when combining with FACS.

A destabilized GFP version expressed together with the *fis* promoter, that is sensitive to fluctuations in substrate availability, was applied to monitor population heterogeneity in GFP synthesis of *E. coli* with ART‐FCM [28]. It was tracked during batch and chemostat phase (D = 0.14 h^−1^) and repeated glucose pulses. During batch phase, GFP fluorescence was correlated to growth rate, whereas in chemostat mode, fluorescence rose unexpectedly. This phenomenon suggests complex physiological regulation mechanisms during bioreactor processes.

The consequences of gradients appearing due to loss in mixing efficiency during up‐scaling, were investigated with an *E. coli* reporter strain expressing GFP together with *rpoS*, which is associated with the general stress response [10]. GFP expression was studied during fed‐batch processes in a STR and a scale‐down bioreactor (STR coupled to a plug flow reactor) simulating extracellular perturbations in substrate concentration and dissolved oxygen level of varying magnitude. It increased during transition from batch to fed‐batch phase, where after it dropped due to dilution effects. In the scale‐down reactor, two subpopulations were observed in response to extracellular perturbations. Thereby, intensity of segregation, as well as the time point of appearance could be related to bioreactor mixing efficiency.

Another chemostat (D = 0.1 h^−1^) study revealed that upon nutrient limitation, populations of *E. coli* and *P. putida* tend to diversify into subpopulations of non‐permeable and permeable cells, which was monitored by automated PI staining [46]. Moreover, against common believes, continuous evolution of the subpopulation ratio in steady state was observed with a stronger effect in *P. putida* than in *E. coli*. In the same setup, various physiological phenomena that influence cell growth and shape and lipid accumulation in *Yarrowia lipolytica* cultures were identified following heterogeneities in SSC and FSC, respectively, staining with Nile red [29].

ART‐FCM could also monitor heterogeneities during scale‐up of fed‐batch processes of CHO cells [152]. To simulate passaging, the culture medium was regularly refreshed, which led to significant variation in proliferation rate. Following changes in FSC and SSC, revealed an increase in the non‐viable subpopulation in early stationary phase. Interestingly, mean cell size of the viable population decreased significantly after inoculation and the first rounds of medium exchange, possibly due to osmotic effects, however, not during subsequent passages.

## 5 AUTOMATED REAL‐TIME FLOW CYTOMETRY FOR AUTOMATED PROCESS CONTROL

When ART‐FCM allows detailed monitoring of physiological changes during bioreactor processes accompanied by automated data analysis, the next step is to establish automated process control. One application is the cytostat [55], where the ART‐FCM is used for feedback control of cell density in a CSTR based on determination of the cell concentration distribution. By means of a control algorithm, the feed and elution rate is increased when the measured cell concentration surpasses a user‐defined set point. Consequently, the culture can be maintained at steady state even at such low cell concentrations that the medium composition is only negligibly changed, making the cell environment precisely defined by the feed composition. The cytostat concept has been applied in several studies evaluating the effect of nutrients, toxic compounds or by‐products on cell growth [55]. For instance, physiological evaluation was performed based on scattering characteristics of *S. cerevisiae* revealing ethanol as the major trigger for cell size increase at critical growth rates [55]. Furthermore, more acetate tolerant *S. cerevisiae* strains with improved production capacities for bioethanol from lignocellulosic hydrolysates were isolated in less than 5 days [53]. The harvest time point was recognized by significant increase in dilution rate on a medium supplemented with acetate.

To advance the cytostat concept, it has been suggested to combine it with genetically encoded reporter strains to enable selection of improved strains based on more complex phenotypic characteristics [37]. Thereby Visualizing Evolution in Real‐Time (VERT) [155, 156] could be adapted, a method to map industrially relevant adaptive phenotypes in microbial populations expanding the knowledge on relevant parameters for strain engineering. VERT has been applied for identification of n‐butanol tolerant *E. coli* phenotypes by visualizing relative proportions of different fluorescently‐labelled cells [156]. It could further be combined with genome shuffling to enhance desired phenotypes or overexpression and deletion studies to reveal the origin of the observed phenotypes and elucidate the underlying tolerance mechanism. The best mutants could be isolated applying ART‐FCM/FACS.

ART‐FCM was also successfully used for automated scale‐up of CHO fed‐batch cultures as well as reliable and reproducible control of the onset of feed addition reaching higher total cell count than respective manual methods [152]. The trigger to initiate feed addition and passage of cells to a larger vessel was an at least four times increase of the non‐viable subpopulation in a culture as this could predict the onset of stationary phase.

Analyzing specific and more complex phenotypes especially based on multi‐parameter fluorescence should be combined with advanced methods for automated data analysis for instance for automated gating (see subsection automated data analysis and reviewed in [23, 78, 157]). Especially, if the ART‐FCM would be further advanced by integration of FACS. Consequently, the determination of new and process‐case‐specific online parameters is of primary importance to use the full potential of ART‐FCM in dedicated feedback control loops [28]. Then also the control strategy has to be advanced, for instance based on detailed process models. These process models can learn from current measurements, improve and forecast process physiology as input for model‐based process control [8]. To our knowledge so far no coupling between ART‐FCM and advanced model‐based process control has been realized. However, existing model‐based process control strategies and soft sensors could be adapted (for instance [158, 159, 160]).

One simple, model‐based process control applying ART‐FCM, termed segregostat, was recently realized [46]. It controls the degree of phenotypic diversification of *E. coli* and *P. putida* populations in CSTR cultures. This novel concept was demonstrated by monitoring membrane permeability in continuous cultures at D = 0.1 h^−1^. Upon nutrient limitation, these cultures tend to diversify into distinct phenotypes, which can be used to trigger automated addition of glucose pulses to maintain a defined degree of diversification. This study sets the foundation for design of advanced process strategies for controlling dynamics in single cell physiology [46].

## 6 CONCLUDING REMARKS

FCM and the underlying technological possibilities have greatly advanced in the past decade. ART‐FCM enables long‐term measurements without missing any important events in bioprocesses and uncovers temporal phenomena that were likely unknown and should be investigated in greater detail [34, 35]. Also more frequent measurements can be performed at a user defined frequency, independent of availability of personnel. Additionally, compared to other methods like omics, that are only partially available on single cell level, multi parameter measurements can easily be established without extended effort of labor and time.

Despite these advantages, ART‐FCM is still rarely applied for bioreactor process monitoring, control and optimization on a laboratory scale and has never been applied in industrial scale. One reason might be that only parts of the ART‐FCM setup are commercially available and might require do‐it‐yourself solutions [20]. Furthermore, the regular user is hindered by the difficulty to properly interface the FC with the process equipment and integrate automated algorithms, soft sensors or process models to handle the resulting multi‐parameter datasets in real‐time [8, 28]. However, if existing algorithms for automated gating and statistical assessments of (sub‐)populations in mono‐ and mixed cultures find their way into FCM analysis as well as digitalization proceeds, this will probably also lead to implementation in automated setups. Then presumably more bioreactor process control applications also in combination with advanced soft sensors will arise, as these are highly dependent on real‐time data analysis. They will enable on‐time feedback regulation of classic process parameters to control microbial physiology. Consequently, once installed, an ART‐FCM will raise the measurement tool FCM to a new level in bioreactor process monitoring (see Figure 1 for a summary of application possibilities).

Moreover, the integration of a possibility to sort during ART‐FCM could further extend bioreactor process monitoring, as it will allow to isolate events of interest for further analysis. Consequently, the demand for deeper investigation of population heterogeneities in bioreactor processes with reporter strains [84], can be fulfilled. However, to our knowledge, such a system does not exist yet. One key factor, hindering the implementation is the missing temperature control within commercialized devices to keep the physical properties of fluids constant and to allow short‐term storage of sorted events. In long‐term perspective, automated sample processing after cell sorting is highly interesting. It would additionally to monitoring cellular dynamics, allow insights, for instance on the proteome of single cells, which would also be a significant step to understand the mechanisms behind population heterogeneity. However, an automated liquid handling platform, if not a complete laboratory automation, would be necessary to process sorted cells in real‐time, which is not expected to be available within the next few years [161, 162].

Lately, growing awareness of advantages associated with miniaturization of analytical devices is pushing forward the progress in designing compact microfluidic devices [163]. The current state of single‐cell analysis involving microfluidics has been reviewed [20, 163–165]. In this context novel highly efficient microfluidics based FCs [153, 166, 167], microfluidic FIA systems [168] and microfluidic fluorescence‐activated droplet sorter [117, 169] are emerging. As comparability with conventional FCM studies could be shown [165, 170], these devices bear great future potential as ART‐FCM on a chip. In this context parallelization might become more relevant, as samples from different bioreactors or different locations inside a bioreactor setup could be analyzed simultaneously.

Many aspects mentioned here might also be adapted to other experimental setups where also fast and reproducible real‐time monitoring of process parameters that are fluorescent is of interest. In conclusion, ART‐FCM will most probably greatly advance in the next years.

## CONFLICT OF INTEREST

The authors declare no conflict of interest.

## AUTHORS' CONTRIBUTIONS

AH is the corresponding author, had the idea for the article, structured and drafted the manuscript. DH contributed to literature search and drafted some chapters. WB supported the development of the concept and critically revised the manuscript. All authors gave consent and read and approved the final manuscript.

## Data Availability

The data that support the findings of this review are available from the corresponding author upon reasonable request.
